# Measures of Spatial Orientation: Spatial Bias Analogs in Visual and Haptic Tasks

**DOI:** 10.1523/ENEURO.0233-22.2022

**Published:** 2022-08-19

**Authors:** Min Jung Kim, Jorge Otero-Millan, Jing Tian, Amir Kheradmand

**Affiliations:** 1 Johns Hopkins University, Baltimore, Maryland 21287; 2 University of California, Berkeley, California, 94720

## Abstract

The primary sensory modality for probing spatial orientation can vary among psychophysical tasks. In the subjective visual vertical (SVV) task, a visual stimulus is used to measure perceived vertical orientation, while a haptic stimulus is used in the subjective haptic vertical (SHV) task. Here we examined disparity in SHV and SVV task results and asked whether it could be related to biases in probing different spatial estimates by each task. Forty-two healthy volunteers (mean ± SD age, 25 ± 10 years; 19 females; 21 left handed) were recruited. The effect of a task to measure spatial orientation was calculated as the difference between SHV and SVV values, and with the head upright and tilted 20° laterally. There was a task bias regardless of head position related to hand use in the haptic task but not handedness (mean head upright ± SEM: left hand, −3.7 ± 1.1°; right hand, 7.9 ± 1.0°). When this task bias was subtracted out, there was a similar spatial bias using each hand in the SHV task that was also comparable to the SVV task (mean head with left tilt: left hand, 3.9 ± 0.7°; right hand, 4.4 ± 0.7°; SVV, 4.9 ± 0.7°; mean head with right tilt: left hand, −4.6 ± 0.9°; right hand, −4.6 ± 0.8°; SVV, −4.7 ± 1.0°). These findings show that the disparity in visual and haptic measures of spatial orientation is primarily related to a modality-specific bias, and once the effect of hand use is removed from the haptic measurements, the spatial bias becomes comparable to the visual task.

## Significance Statement

The brain has to account for the head and eye positions to maintain spatial orientation. Accordingly, a difference in visual and haptic measurements of spatial orientation may raise the question of whether each task measures a different spatial estimate or whether their disparity is primarily related to a measurement bias in each task. Our findings show a modality-specific measurement bias in each task that, once removed, made the measures of spatial orientation comparable between both tasks.

## Introduction

Spatial orientation is a multisensory process that generates a coherent perception of self with respect to the surrounding environment. This multimodal function can be studied using the direction of gravity as a reference to measure orientation in space (for review, see Kheradmand and Winnick, 2017). The primary sensory modality for probing spatial orientation can vary among psychophysical tasks. In the subjective visual vertical (SVV) task, a visual stimulus (e.g., a visual line) is used to measure perceived vertical orientation, while a haptic stimulus (e.g., a metal bar) is used in the subjective haptic vertical (SHV) task (Kheradmand and Winnick, 2017). With such differences in probing spatial orientation, understanding inherent biases in each task is critical to reliably compare and interpret the findings from these methods.

Prior studies have shown disparity in visual and haptic measures of spatial orientation, raising the question of whether SHV and SVV access different sensory estimates or whether there is a modality-specific bias in each task related to different methodologies (Guerraz et al., 2000; Lejeune et al., 2004; Bortolami et al., 2006; Tarnutzer et al., 2012a; Fraser et al., 2015). Since visual inputs are used in the SVV task, the brain must account for both the head and eye positions to estimate the orientation of the visual stimulus (De Vrijer et al., 2009; Kheradmand and Winnick, 2017). In the SHV task, tactile and proprioceptive information is used to estimate the orientation of the haptic stimulus. Therefore, SVV and SHV tasks each rely on sensory inputs encoded in different reference frames, and their disparity might be related to how the brain estimates the position of the eye in the SVV task versus the position of the hand in the SHV task.

A previous study found that SHV tasks were directly affected by a hand bias (Kim et al., 2022). This bias was in the same direction of the hand use with a leftward bias when SHV was measured with the left hand, and a rightward bias when it was measured with the right hand. SHV was also biased by the left-side or right-side placement of the haptic bar, showing that in addition to the hand-in-body position (i.e., the laterality effect), the hand-in-space position also influenced haptic responses. The hand bias was still present when the orientation of the haptic bar was reported relative to a visual line showing that the bias was primarily related to the haptic nature of the task and not confined to the perception of gravity. There was no effect from handedness, and similar hand biases were found in the haptic task in both left-handers and right-handers. These findings show that using the hand in the SHV task can affect the measurement of spatial orientation and that such modality-specific biases should be considered when the results are compared between the SHV and SVV tasks.

In the present study, we examined the disparity in SHV and SVV results and asked whether it can be related to a measurement bias in each task. This modality-specific bias was removed by subtracting measurements at two different head positions to derive the spatial bias in each task. Since the brain has to account for the eye position in the visual task and the hand position in the haptic task, a difference in spatial bias measured by the visual and haptic tasks would suggest that their disparity is primarily related to a measurement bias between the tasks. Also, if the hand bias is a fixed measurement bias in the haptic task, when it is removed from the SHV measurements, there should be no difference in the haptic vertical estimates using each hand or with a change in handedness. To address these questions, here we implemented a forced-choice paradigm where the haptic stimulus is touched at each trial to determine its orientation relative to perceived vertical orientation. A similar paradigm was used for SVV measurement and for comparison with SHV results.

## Materials and Methods

### Participants

The experiments were approved by the Johns Hopkins University institutional review board, and informed written consent was obtained from all participants. Forty-two healthy volunteers (mean ± SD age, 25 ± 10 years; 19 females) participated in the study, and they were all reported to be in good health without visual, vestibular, neurologic, or psychiatric illness. Of the 42 participants, 21 were left handed (age, 25 ± 12 years; 11 females) and 21 were right handed (age, 24 ± 10 years; 8 females). Subjects completed the Edinburgh Handedness Inventory and the Tapley-Bryden proficiency test before the experiment to quantify their handedness (Oldfield, 1971; Salmaso and Longoni, 1985; Tapley and Bryden, 1985). In the Edinburgh Handedness Inventory, subjects indicated which hand they preferred to use daily in a questionnaire. In the Tapley and Bryden proficiency test, subjects are asked to mark dots in circles as quickly as possible based on their hand preferences in 20 s intervals, for four trials, to verify handedness measurement.

### Experimental procedures

Two different tasks were used to measure the perception of spatial orientation including SHV and SVV tasks. Both tasks measured the perceived gravitational vertical using a haptic stimulus in SHV task and a visual stimulus in the SVV task. Subjects were in complete darkness to eliminate all visual cues other than the visual stimulus in the SVV task.

#### Subjective haptic vertical

We use a method described previously for SHV measurement (Kim et al., 2022). The haptic stimulus was a metal bar that consisted of two pieces placed 30 cm in front of the subject’s hand. The haptic bar was rotated to specific orientations at each trial and an auditory signal (a double beep at 1000 Hz) indicated that the motor had finished moving. The center of the bar was aligned with a subject’s hand so that they could comfortably extend the forearm while resting the elbow on a flat armrest. Subjects were instructed to touch the two edges of the haptic bar with a pinch grip only using the tips of their thumb and index finger. With the opposite hand, they reported whether the stimulus was tilted to the left or right of perceived vertical orientation by clicking the left or right button of a computer mouse. As soon as the subjects provided a response after retracting their hand from the haptic bar, the next trial began with another auditory signal (a single beep at 1000 Hz) to indicate that the motor was starting to move. There was a window of 10 s to feel the stimulus and respond at each trial. If the response time was longer, the trial was aborted and a new trial started after a button was clicked.

**Figure 1.**
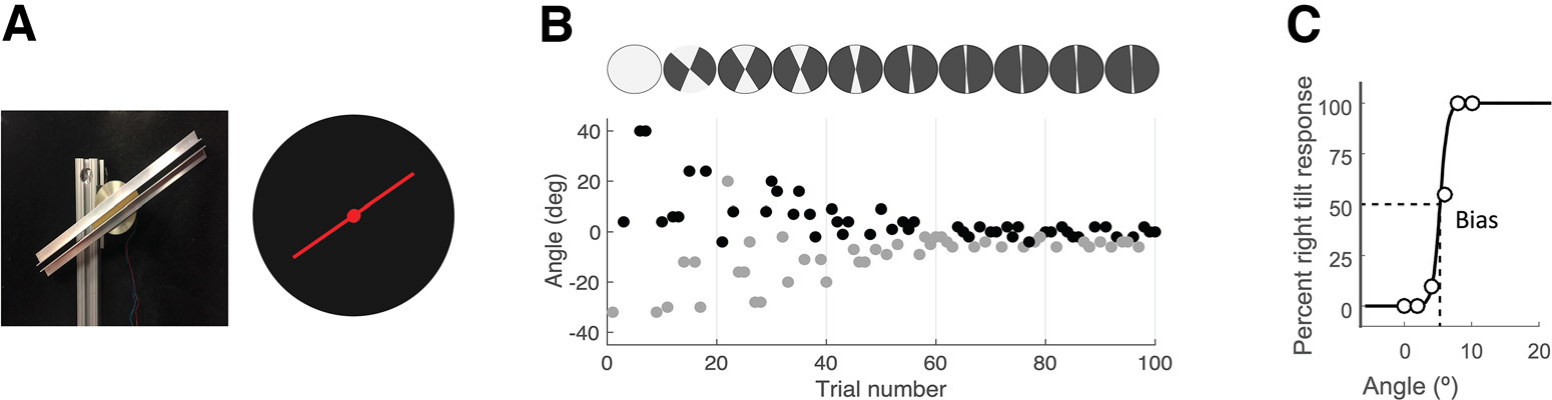
***A***, The haptic stimulus in the SHV task was a bar that consisted of two pieces with parallel edges mounted on a stepper motor, and the visual stimulus was a red line in the SVV task. In both tasks, the orientation of the stimulus was reported with respect to the perceived vertical. ***B***, Sample time course of 100 trials with responses from the paradigm used in both tasks. The *y*-axis shows the angle of the stimulus presented, and the color indicates the response for that trial (left, in gray; right, in black). The stimulus angles were presented randomly within a range that started at 180° and then was adjusted based on previous responses (gray sectors in the top circles). ***C***, An example of psychometric fit to the responses in the paradigm. The bias value (SVV/SHV) is determined as the center of the curve (i.e., point of subjective equality), where the probabilities of right and left responses are equal. Precision is determined as the slope of the curve.

The haptic bar was 26 cm long, and each piece had an edge of 1.5 mm wide and 1.4 cm thick with a gap of 7 mm between the two pieces (Fig. 1*A*). The center of the bar was mounted on a stepper motor with a holding torque of 0.26 Nm and a step size of 1.8° (Stepperonline). An MPU-9250 accelerometer sensor was attached to the haptic bar. Both the stepper motor and accelerometer sensor were connected to an Arduino Uno board and controlled by an Arduino–MATLAB interface (Arduino). The stepper motor allowed the bar to rotate in the roll plane. The accelerometer recorded the angle of the haptic stimulus, relative to gravitational vertical with a ± 0.1° accuracy. The stepper motor moved to adjust the haptic stimulus based on the difference between the accelerometer reading of the angle and the target angle given by the paradigm, with ±1.25° accelerometer-to-software error permitted for each trial. Thus, if there was an error produced by the motor or the angle of the haptic bar was changed while touching it, the trial was aborted, and the paradigm advanced to the next trial. In such cases, the missed angle was presented again later by the paradigm as another trial.

#### Subjective visual vertical

We used a method described previously for SVV measurement (Kim et al., 2022). In each trial, a red visual line (length, 7.6° of visual angle; width, 0.13° of visual angle) was presented in random angles with a step size of 2° around a red fixation dot at eye level (diameter, 0.33°; distance, 135 cm) in front of the subject. The task was to click a left or right button using a controller bimanually to report whether the upper half of the line was tilted to the left or right of the perceived vertical orientation. The fixation dot appeared first for 1 s, after which the visual line was presented for a minimum of 300 ms and maximum of 1.5 s until the subject responded. If the response time became longer, the trial was aborted and a new trial was started after a button was clicked. In such cases, the missed angle was presented again later by the paradigm as another trial.

### Adaptive two-alternative forced-choice paradigm

We used a custom paradigm written in MATLAB using Psychtoolbox (Kleiner et al., 2007) to present the stimuli and measure responses in the SHV and SVV tasks. This adaptive paradigm is described in detail in previous studies, as depicted in Figure 1*B* (Otero-Millan and Kheradmand, 2016; Winnick et al., 2019; Kim et al., 2022). For both SVV and SHV tasks, the paradigm started with presenting the stimulus at random angles within the range of 180°, but the range of angles was adjusted at every block of 10 trials as the paradigm continued. The new range was centered around the SVV/SHV value calculated from the responses in the previous 30 trials (see the SVV/SHV calculation described in the subsection Data analysis) and was decreased by half until the paradigm reached the seventh block, after which it remained constant at 8° for the remaining trials. Thus, the paradigm could adapt and track spatial perception and was not biased by only providing a fixed set of angles to measure the perceived vertical in the haptic and visual tasks.

### Experiment setup

In both tasks, subjects sat upright in a completely dark room that was designed to perform experiments in the dark with all walls, floor, and ceiling in black color and light sealed. In the SVV task, the line stimulus appeared at the center of a CRT monitor (1280 by 1024 pixels) that was covered by black cardboard with a circular opening (diameter, 28 cm). To further eliminate any potential cues from the CRT monitor, the brightness and contrast levels of the screen were set to the minimum at a luminance of <0.5 cd/m^2^. The height of the CRT monitor was adjusted to align the center of the monitor with the eye level for each subject.

In both SVV and SHV tasks, the head was immobilized using a molded bite bar. Subjects were monitored remotely through an infrared camera to ensure that they remained on the bite bar during the experiment and were following instructions during the task. They were instructed to wear their glasses if needed for the SVV task. The SVV and SHV tasks were completed in two sessions on separate days. In each session, SHV was measured using either the left or right hand while the head was upright or tilted 20° laterally to the left or right shoulder (100 trials at each head position). Trials of three different head positions were performed consecutively. The following session was similar except that the opposite hand was used for SHV measurement. Three sets of SVV recordings at each head position were performed in both sessions for comparison with the SHV measurements. A set of 100 trials of the SVV task lasted 2.9 min ± 1 s (mean ± SEM), while a set of 100 trials of the SHV task lasted 5.8 min ± 5 s. The order of the sessions, head tilts, and the tasks during each session was counterbalanced among subjects. The haptic bar was placed on the right side of the subject for SHV measurement with the right hand and it was placed on the left side of the subject for SHV measurement with the left hand.

### Data analysis

Using data collected from the two-alternative forced-choice paradigm in each task, a psychometric curve was fitted to the 100 trial responses using a cumulative Gaussian function (MATLAB fitglm with probit link function) and a generalized linear regression model (Fig. 1*C*). With this method, the final SVV or SHV value was determined as the angle at which the probability of a left or right response was 50% in their corresponding tasks (point of subjective equality). SVV or SHV precision was calculated as the angle difference between the 50% and 75% points on the psychometric fits.

The effect of the task used to measure vertical perception (i.e., task bias) was calculated as the difference between SHV and SVV values from the same recording session in each subject, as follows: (SHV−SVV). The spatial bias was measured in each task as the difference in the values between the head upright and tilted laterally (i.e., 20° left/right), as follows: $\left({\text{SHV}}_{\text{tilt}}-{\text{SHV}}_{\text{upright}}\right)$ and $\left({\text{SVV}}_{\text{tilt}}-{\text{SVV}}_{\text{upright}}\right)$. When the head is tilted, the eyes partially roll in the opposite direction (Otero-Millan and Kheradmand, 2016). As a result, the eye position during head tilt is not similar to when the head is upright. In the haptic task, however, the effect of hand position does not change with head tilt. Therefore, the task difference between SVV and SHV can yield the effect of eye-in-head position in each subject (i.e., visuospatial bias), as follows: $\left({\text{SVV}}_{\text{tilt}}–{\text{SVV}}_{\text{upright}}\right)–\left({\text{SHV}}_{\text{tilt}}–{\text{SHV}}_{\text{upright}}\right)$.

Repeated-measures ANOVA was run in MATLAB to compare the results between different tasks (SVV/SHV), hand use (left/right), handedness (left handed/right handed), and head position (left tilt/upright/right tilt). To calculate correlations across subjects, we used the Pearson method to obtain the coefficient.

## Results

The mean SHV values using the right and left hand and the corresponding SVV values are summarized in Table 1. The SVV values measured in the same session along with the left-hand SHV were 3.9 ± 1.1° (mean ± SEM) with the left head tilt, −0.4 ± 0.4° with the head upright, and −6.0 ± 1.3° with the right head tilt, and they were 4.9 ± 1.0°, −0.5 ± 0.4°, and −4.3 ± 1.3°, respectively, when measured in the same session along with the right-hand SHV. These results are consistent with a bias opposite to the tilt direction that represents overestimation of vertical orientation at small head tilt angles and is known as the E-effect (Tarnutzer et al., 2009b; Kheradmand and Winnick, 2017). There was no difference between the SVV tasks from these two sessions, while the head position had a significant effect (two-way repeated-measures ANOVA; SVV session, *p* = 0.08, head position, *p* < 0.001).

**Table 1:** SHV and SVV Results with negative values as leftward bias and positive values as rightward bias

Head	Hand used	SHV ± SEM (°)	SVV ± SEM (°)		Task bias ± SEM (°)	SHV spatial bias ± SEM (°)	SVV spatial bias ± SEM (°)	
Left tilt	Left hand	−0.2 ± 1.1	3.9 ± 1.1	4.4 ± 0.7	−4.2 ± 1.5	3.9 ± 0.7	4.3 ± 1.0	4.9 ± 0.7
	Right hand	11.8 ± 0.9	4.9 ± 1.0		6.9 ± 1.3	4.4 ± 0.7	5.4 ± 0.9
Upright	Left hand	−4.2 ± 1.1	−0.4 ± 0.4	−0.5 ± 0.3	−3.7 ± 1.1			
	Right hand	7.4 ± 1.0	−0.5 ± 0.4		7.9 ± 1.0			
Right tilt	Left hand	−8.8 ± 0.9	−6.0 ± 1.3	−5.2 ± 0.9	−2.8 ± 1.5	−4.6 ± 0.9	−5.6 ± 1.4	−4.7 ± 1.0
	Right hand	2.8 ± 1.0	−4.3 ± 1.3		7.2 ± 1.5	−4.6 ± 0.8	−3.8 ± 1.3

The task bias was calculated as the difference between the SHV task and the SVV task for each subject. The spatial bias was calculated as the difference between SHV or SVV task values with the head tilted and upright.

**Figure 2.**
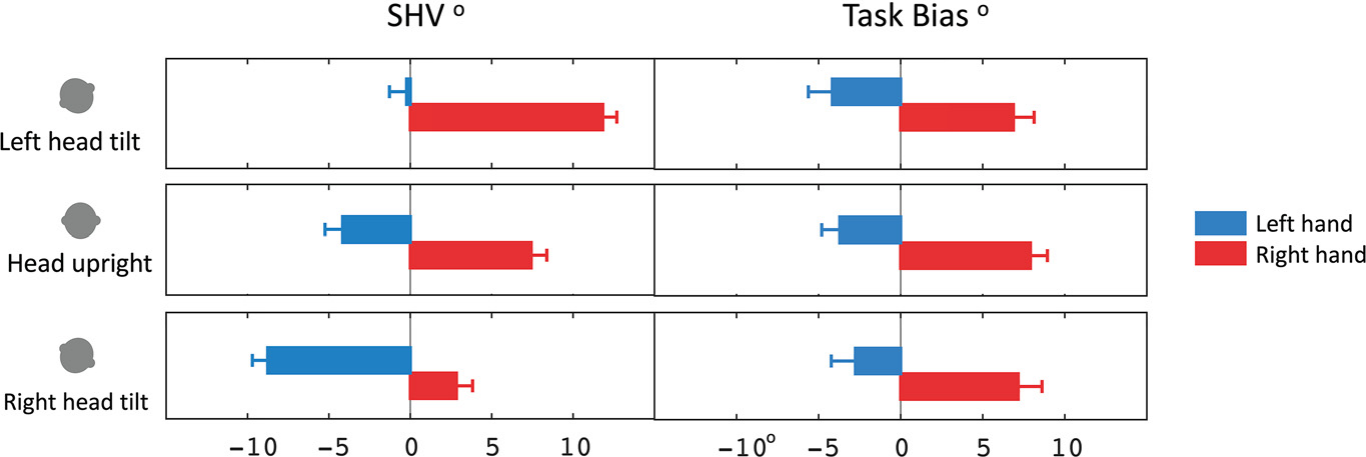
Raw SHV value and the task bias are shown for each hand use and three different head tilt positions. Error bars show the SEM.

The mean left hand SHV values were −0.2 ± 1.1° with the left head tilt, −4.2 ± 1.1° with the head upright, and −8.8 ± 0.9° with the right head tilt. The mean right-hand SHV values were 11.8 ± 0.9° with the left head tilt, 7.4 ± 1.0° with the head upright, and 2.8 ± 1.0° with the right head tilt (Fig. 2, left). There was a significant effect of hand use and head tilt position but no significant interaction between the hand use and head tilt position (two-way repeated-measures ANOVA; factor of hand, *p* < 0.001; factor of head, *p* < 0.001; interaction of hand and tilt, *p* = 0.87).

### Task bias

The task bias was calculated as the difference between SHV and SVV task values for each subject (Fig. 2, right). The task bias for the left-hand SHV task was −4.2 ± 1.5° with the left head tilt, −3.7 ± 1.1° with the head upright, and −2.8 ± 1.5° with the right head tilt (Table 1). The task bias for the right-hand SHV was 6.9 ± 1.3° with the left head tilt, 7.9 ± 1.0° with the head upright, and 7.2 ± 1.5° with the right head tilt (Table 1). The task bias was significantly affected by the hand use but not by the head tilt position (two-way repeated-measures ANOVA: factor of hand, *p* < 0.001; factor of head, *p* = 0.80). These results show a consistent task bias between SHV and SVV task measurements across all head tilt positions.

### Spatial bias

**Figure 3.**
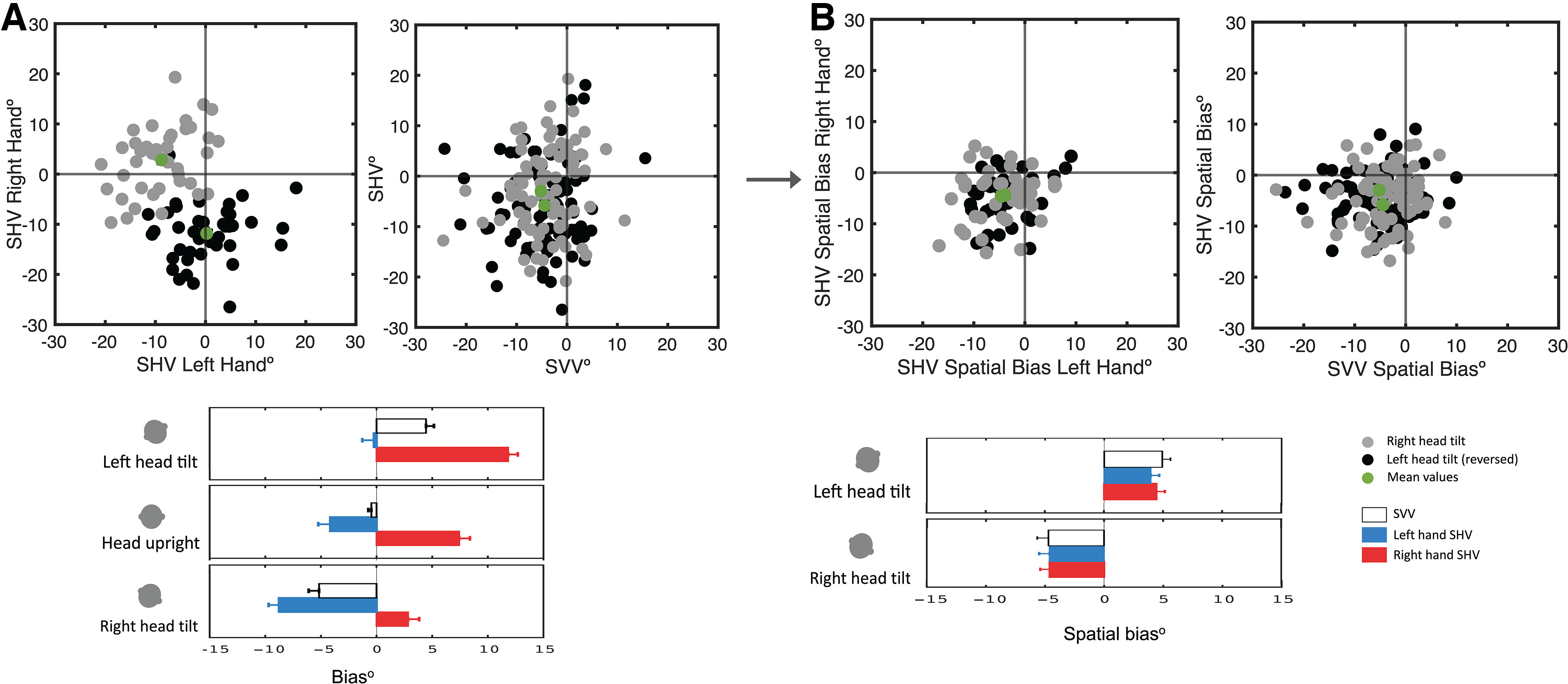
***A***, individual SHV and SVV measurements are shown for both head tilts on the top plots (left tilt reversed) with the group means (green). The group means for the SHV task with each hand, and those for the SVV task are also shown on the bottom plots (error bars show the SEM). ***B***, SHV and SVV spatial bias for both head tilts are shown on the top plots (left tilt reversed) with the group means (green). The group means of SVV and SHV spatial biases are shown on the bottom plots. These results show that once the effect of hand bias is removed, the SHV spatial biases measured with each hand are similar, and they also conform with the SVV spatial bias.

The effect of hand bias was removed by subtracting the SHV task measurement with the head upright from the head tilted (i.e., spatial bias; Fig. 3, Table 1). The spatial bias for SHV with the left head tilt was 3.9 ± 0.7° using the left hand, and 4.4 ± 0.7° using the right hand. The spatial bias for SHV task with the right head tilt was −4.6 ± 0.9° using the left hand and −4.6 ± 0.8° using the right hand. The spatial bias was also calculated for the SVV task, which was 4.9 ± 0.7° with the left head tilt and −4.7 ± 1.0° with the right head tilt. The spatial bias was significantly different among the tilt positions regardless of the task (two-way repeated-measures ANOVA; factor of tilt, *p* < 0.001; factor of hand/task, *p* = 0.92). These results show that when the hand bias is removed, the spatial bias is similar using each hand in the SHV task or across the two tasks of SHV and SVV (Fig. 3).

### Visuospatial bias

**Figure 4.**
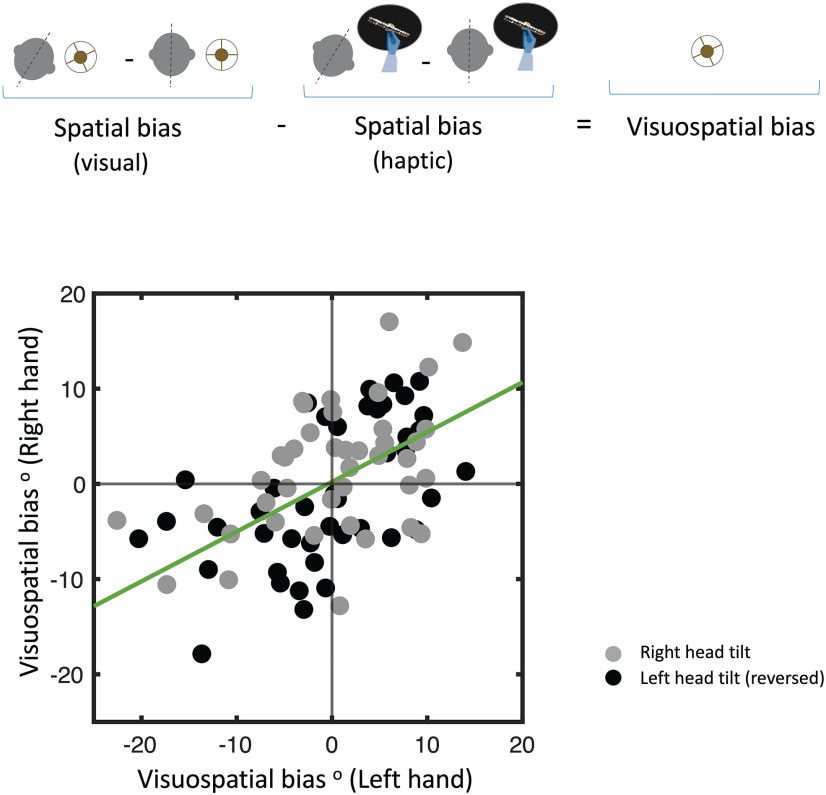
The effect of hand use in the haptic task can be removed when the measurements with the head upright are subtracted out from those with the head tilted. In the visual task, however, the change in eye position with head tilt cannot be subtracted out. Accordingly, a difference between SHV and SVV tasks can provide the visuospatial bias in each subject. There is a significant correlation (bottom graph) between the visuospatial biases measured using each hand in the haptic task (Pearson’s correlation: *r*^2^ = 0.38, *p* < 0.001).

The effect of eye-in-head position in the SVV task or the visuospatial bias was calculated as the difference between the spatial biases in SVV and SHV tasks (Fig. 4). The visuospatial bias with the left head tilt was 0.5 ± 1.3° when the left hand was used for the haptic task, and it was 1.0 ± 1.1° when the right hand was used. The visuospatial bias with the right head tilt was −1.0 ± 1.6° when the left hand was used, and 0.7 ± 1.3° when the right hand was used. These results show a spatial bias between visual and haptic tasks related to the effect of eye-in-head position. There was a significant correlation between these visuospatial biases derived from using each hand in the haptic task (Fig. 4; Pearson’s correlation: *r*^2^ = 0.38, *p* < 0.001).

### Effect of handedness

The Edinburgh handedness laterality quotients obtained for each participant ranged from −100 to 100, with negative numbers representing left handedness and positive numbers representing right handedness. The handedness laterality quotient for the left-handers was −78 ± 18 (mean ± SD), and 75 ± 20 for the right-handers. The range of Tapley and Bryden laterality quotients of the participants was −0.34 to 0.40, with negative numbers representing left-handedness and positive numbers representing right handedness. The mean Tapley and Bryden laterality quotient for the left-handers was −0.19 ± 0.10, and 0.24 ± 0.09 for the right-handers. The two laterality quotients were highly correlated across subjects (*r*^2^ = 0.85).

The left-hand SHV values were 0.2 ± 1.6° with the left head tilt, −3.7 ± 1.7° with the head upright, and −9.2 ± 1.4° with the right head tilt for the left-handers, and they were −0.7 ± 1.4° with the left head tilt, −4.6 ± 1.4° with the head upright, and −8.3 ± 1.3° with the right head tilt for the right-handers. The right-hand SHV values were 11.2 ± 1.2° with the left head tilt, 6.7 ± 1.4° with the head upright, and 2.6 ± 1.3° with the right head tilt for the left-handers, and they were 12.5 ± 1.3° with the left head tilt, 8.1 ± 1.3° with the head upright, and 3.1 ± 1.5° with the right head tilt for the right-handers (Table 2).

**Table 2:** SHV Bias per head tilt position, hand use, and handedness

Head		SHV ± SEM (°)	
		Left handed	Right handed
Left tilt	Left hand	0.2 ± 1.6	−0.7 ± 1.4
	Right hand	11.2 ± 1.2	12.5 ± 1.3
Upright	Left hand	−3.7 ± 1.7	−4.6 ± 1.4
	Right hand	6.7 ± 1.4	8.1 ± 1.3
Right tilt	Left hand	−9.2 ± 1.4	−8.3 ± 1.3
	Right hand	2.6 ± 1.3	3.1 ± 1.5

**Figure 5.**
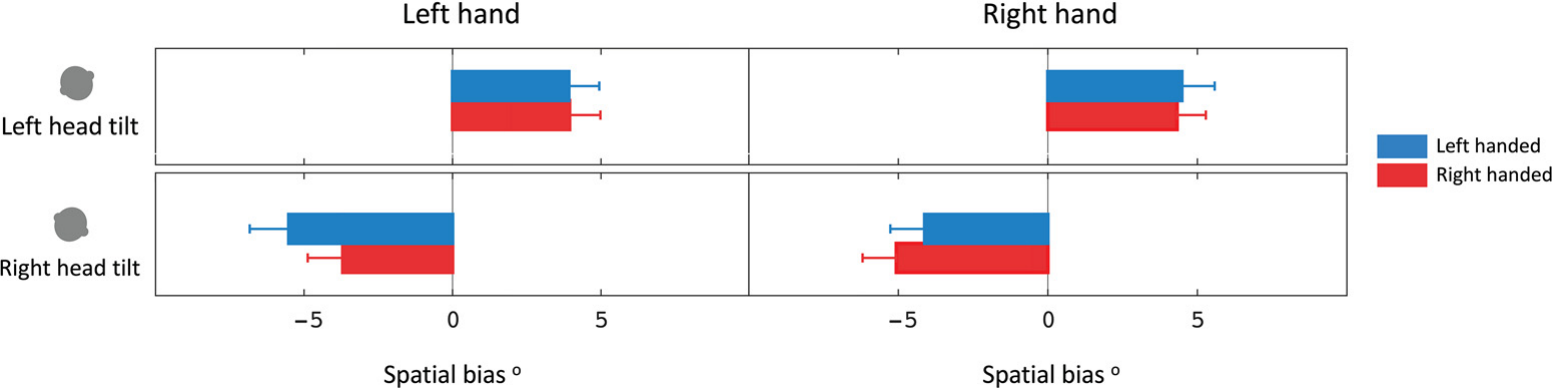
Mean spatial bias measured with each head tilt separated by hand use in right-handers and left-handers. Error bars show the SEM.

There was a significant effect of head tilt position and hand used but not handedness in the SHV task (Figs. 2 & 5; three-way repeated-measures ANOVA; factor of head, *p* < 0.001; factor of hand, *p* < 0.001; factor of handedness, *p* = 0.78). Similarly, the effect of handedness was not significant in the SVV task data (factor of handedness, *p* = 0.22).

### Precision

To measure variability in SHV and SVV measurements, precision was calculated as the slope of the corresponding psychometric curves (Table 3). The mean SHV precision was 3.8 ± 0.2° with the left head tilt, 3.6 ± 0.2° with the head upright, and 3.7 ± 0.2° with the right head tilt. There was no significant effect of head tilt position, hand use, or handedness on the SHV precision (three-way repeated measures ANOVA; factor of tilt, *p* = 0.64; factor of hand, *p* = 0.97; factor of handedness, *p* = 0.27). The mean SVV precision was 2.1 ± 0.1° with the left head tilt, 1.1 ± 0.1° with the head upright, and 2.1 ± 0.1° with the right head tilt. Overall, the SVV task was significantly more precise than the SHV task, and there was an effect of head tilt position on the SVV task with more precise responses when the head was upright than tilted (two-way repeated-measures ANOVA; factor of task, *p* < 0.001; factor of tilt, *p* < 0.001; task and tilt interaction, *p* < 0.001).

**Table 3:** SHV and SVV Precision results per head tilt position, hand use, and handedness

Head	SHV ± SEM (°)				SVV ± SEM (°)
Left tilt	3.8 ± 0.2	Left hand	3.7 ± 0.3	3.6 ± 0.4 (left handed)	2.1 ± 0.1
				3.7 ± 0.5 (right handed)	
		Right hand	3.9 ± 0.3	3.8 ± 0.4 (left handed)	
				3.9 ± 0.5 (right handed)
Upright	3.6 ± 0.2	Left hand	3.7 ± 0.3	3.4 ± 0.3 (left handed)	1.1 ± 0.1
				4.0 ± 0.6 (right handed)	
		Right hand	3.5 ± 0.2	3.2 ± 0.3 (left handed)	
				3.8 ± 0.4 (right handed)
Right tilt	3.7 ± 0.2	Left hand	3.7 ± 0.3	3.6 ± 0.3 (left handed)	2.1 ± 0.1
				3.9 ± 0.4 (right handed)	
		Right hand	3.8 ± 0.3	3.2 ± 0.3 (left handed)	
				4.4 ± 0.5 (right handed)

## Discussion

In this study, we examined the disparity in accuracy (bias) and precision (variance) of two spatial orientation tasks: SHV and SVV. The results show a task bias between SHV and SVV responses. In the haptic task, the task bias was related to the hand use with a consistent difference between the left-hand and right-hand measurements regardless of the head position. When the task bias was subtracted out from the haptic responses, there was a similar spatial bias in the haptic task that was comparable to the visual task. These findings show that the disparity in visual and haptic measures of spatial orientation is primarily related to a measurement bias, and once the effect of hand use is removed from the haptic measurements, the spatial bias becomes comparable.

Our results show that the haptic task bias is a fixed bias regardless of the head position, and it does not represent a change in spatial estimate. In line with this finding, SHV bias was different when using each hand. Once this hand bias was subtracted out, the spatial bias induced by head tilt was comparable between responses from both hands. In addition, in support of a sensory nature of the hand bias, there was no effect from handedness, and both left-handers and right-handers showed a consistent laterality bias related to using the left hand or right hand in the haptic task. Previous studies have also shown the effect of task bias related to hand use in the SHV task (Bauermeister et al., 1964; Tarnutzer et al., 2012b; Kim et al., 2022). The bias could be related to the haptic nature of the task, and how the brain must use sensory information about the hand-in-body (left vs right hand) and hand-in-space to estimate the positions of the hand and haptic stimulus.

The discrepancy in SVV and SHV task results can also be attributed to different sensory processes in each task (Kheradmand and Winnick, 2017). When visual cues are removed, the brain must rely on signals that encode the position of the head and body in space to determine the orientation of external stimuli. In the SVV task, the position of the eye in the orbit can affect perceived orientation of the visual stimulus. With a lateral head tilt, there is a compensatory movement of the eyes in the opposite direction, which is less than the actual amount of head tilt, and is known as the ocular counter-roll (Otero-Millan and Kheradmand, 2016). With this low-gain response, the vertical meridians of the eyes deviate from the axis of gravity, and the orientation of the image changes on the retina. This physiological constraint can result in a visual bias that contributes to systematic errors in SVV responses during head tilt (Mast and Jarchow, 1996; Van Beuzekom and Van Gisbergen, 2000; De Vrijer et al., 2009; Tarnutzer et al., 2009a). SHV responses also become less accurate with head tilt, but the pattern of error can be different from SVV responses, which could be because of different task biases related to hand use in the haptic task and eye position in the visual task (Bauermeister et al., 1964; Luyat et al., 2001; Gentaz et al., 2008; Fraser et al., 2015). Because of the low gain of ocular counter-roll, the eye position during head tilt is not similar to when the head is upright, and as a result the effect of eye-in-head position cannot be removed from the SVV spatial bias. In the SHV task, however, since the effect of hand use does not change with head tilt, the hand bias can be removed from the haptic spatial bias. Our results suggest that after removing the hand bias from the haptic responses, the task bias between SVV and SHV measurements could be related to this effect of eye-in-head position (i.e., the visuospatial bias; Fig. 4). Here we did not directly measure ocular counter-roll, and future studies will have to examine whether this visuospatial bias is correlated with the actual or the brain estimate of the eye position within the orbit.

The direction of spatial bias induced by head tilt usually depends on the angle of the tilt. At large angles (e.g., >60°), the bias is generally in the same direction of the head tilt position, which represents underestimation of vertical orientation (known as the Aubert or A-effect; Tarnutzer et al., 2009b; Kheradmand and Winnick, 2017). At smaller angles (e.g., <60°); however, the spatial bias can be in the opposite direction of the head tilt position, which represents overestimation of vertical orientation (known as the E-effect; Tarnutzer et al., 2009b; Kheradmand and Winnick, 2017). Based on our results, the effect of hand use changed the direction of haptic vertical responses at the small tilt angle of 20° (Fig. 2). Once the effect of hand bias was removed from the haptic measurements, the direction of spatial bias induced by head tilt conformed with the one measured in the visual task (Fig. 3). These results show the importance of accounting for the task bias when spatial estimates are compared across SHV and SVV tasks.

Here we used a forced-choice paradigm instead of using a method with manual adjustment of the stimulus to perceived vertical orientation. With manual adjustment, a motor bias can result in a systematic shift or hysteresis from moving the stimulus (e.g., a rightward bias with rightward movement of the stimulus; Bauermeister et al., 1964; Schuler et al.,2010; Tarnutzer et al., 2012b). Also, with manual haptic adjustments, SHV measurements with the right hand can become biased to the left side, while adjustments with the left hand can become biased to the right side (Bauermeister et al., 1964). In addition, for both visual and haptic stimuli, our measurement paradigm used a minimum angle size within a range of 2° as perceived vertical responses are normally within 2° of earth vertical (Kheradmand and Winnick, 2017). In the SHV task, a minimum step size of 1.8° was used for the haptic bar, and a minimum step size of 2° was used for the visual line in the SVV task. Therefore, the step size used to present haptic and visual stimuli were comparable between both tasks, and it could not affect the resolution of SHV and SVV measurements.

The higher sensory noise during head tilt can also cause larger variability of spatial estimates compared with when the head is upright (De Vrijer et al., 2009). Such variability is reflected in the precision of SHV and SVV measurements at different head tilt positions (Luyat et al., 2001; Gentaz et al., 2008; Schuler et al., 2010; Barra et al., 2012; Fraser et al., 2015). Our results are consistent with an overall higher variability in SHV results than in SVV results. In the visual task, head tilt increased the variability of responses, but such an effect of head position at the small tilt angle of 20° was masked by the effect of hand bias in the haptic task.

In conclusion, the present results show measurement biases related to hand use in the haptic vertical task and eye-in-head position in the visual vertical task. The task bias was consistent regardless of head position, and when it was subtracted out, there was a similar spatial bias using each hand in the haptic task that also conformed with the spatial bias in the visual task. These findings show that the disparity in visual and haptic measures of spatial orientation is primarily related to a modality-specific bias in each task.

## Synthesis

Reviewing Editor: Christine Portfors, Washington State University Decisions are customarily a result of the Reviewing Editor and the peer reviewers coming together and discussing their recommendations until a consensus is reached. When revisions are invited, a fact-based synthesis statement explaining their decision and outlining what is needed to prepare a revision will be listed below. The following reviewer(s) agreed to reveal their identity: Alexander Tarnutzer.

This is a well conceived study that follows up on previous publications in the field. The manuscript would benefit from a more detailed discussion of the role of ocular counterroll (which was not measured in the current study in participating subjects) in this modality specific bias and reviewing more thoroughly previous publications in the field with regards to methodological differences (e.g. positioning of bar).

Specific comments

Abstract:

Page 1: “When this task bias was subtracted out, there was a similar spatial bias using each hand in SHV task that was comparable with SVV task (left tilt: left hand 3.9 {plus minus} 0.7{degree sign}, right hand 4.4 {plus minus} 0.7{degree sign}, SVV 4.9 {plus minus} 0.7; right tilt: left hand -4.6 {plus minus} 0.9{degree sign}, right hand -4.6 {plus minus} 0.8{degree sign}, SVV -4.7 {plus minus} 1.0).”

There is no mentioning of roll-tilted positions before in the abstract. thus this result comes out of the blue. please provide tilt-angles in the methods section

Introduction:

Material and methods:

Page 4: SHV: “The center of the bar was mounted on a stepper motor with a holding torque of 0.26 Nm and a step size of 1.8{degree sign} (Stepperonline).”

As all adjustments will be passive, the step size of 1.8{degree sign} of the stepper motor limits the roll resolution of the haptic bar considerably. This needs to be addressed in the discussion section (limitations).

Page 4: I would like to see a picture of the haptic bar in addition to the graphical illustration in Fig 1A

Results:

Page 6: Figure 2 - Interestingly, the task bias was larger when using the right hand compared to the left hand. was this difference statistically significant?

Page 7 - Table 1: At 20deg ear down position over-compensation for roll-tilt is observed. this might confuse the reader at first and thus should be discussed (E-effect).

Page 7 - Figure 3: Here SD are used, while on most other places SEM are applied. Please explain why different approaches were chosen to indicate the variabilty of data points within subjects. I suggest to consistently use either SD or SEM.

Discussion:

Page 11: “Such variability is reflected in precision of SHV and SVV measurements at different head tilt positions (Bauermeister et al., 1964; Luyat et al., 2001; Gentaz et al., 2008; Barra et al., 2012; Fraser et al., 2015).”

Here the authors may include the citation by Schuler et al. that describes the variability in SHV adjustments in the roll plane (Schuler JR, Bockisch CJ, Straumann D, Tarnutzer AA. Precision and accuracy of the subjective haptic vertical in the roll plane. BMC Neurosci. 2010 Jul 14;11:83. doi: 10.1186/1471-2202-11-83. PMID: 20630097; PMCID: PMC2912915.)

General comment: It would be helpful to have a limitations section.
